# Automated Region Extraction from Thermal Images for Peripheral Vascular Disease Monitoring

**DOI:** 10.1155/2018/5092064

**Published:** 2018-12-13

**Authors:** Jean Gauci, Owen Falzon, Cynthia Formosa, Alfred Gatt, Christian Ellul, Stephen Mizzi, Anabelle Mizzi, Cassandra Sturgeon Delia, Kevin Cassar, Nachiappan Chockalingam, Kenneth P. Camilleri

**Affiliations:** ^1^Centre for Biomedical Cybernetics, University of Malta, Msida MSD2080, Malta; ^2^Department of Podiatry, University of Malta, Msida MSD2080, Malta; ^3^Department of Surgery, University of Malta, Msida MSD2080, Malta; ^4^Faculty of Health Sciences, Staffordshire University, Staffordshire ST4 2DE, UK

## Abstract

This work develops a method for automatically extracting temperature data from prespecified anatomical regions of interest from thermal images of human hands, feet, and shins for the monitoring of peripheral arterial disease in diabetic patients. Binarisation, morphological operations, and geometric transformations are applied in cascade to automatically extract the required data from 44 predefined regions of interest. The implemented algorithms for region extraction were tested on data from 395 participants. A correct extraction in around 90% of the images was achieved. The process of automatically extracting 44 regions of interest was performed in a total computation time of approximately 1 minute, a substantial improvement over 10 minutes it took for a corresponding manual extraction of the regions by a trained individual. Interrater reliability tests showed that the automatically extracted ROIs are similar to those extracted by humans with minimal temperature difference. This set of algorithms provides a sufficiently accurate and reliable method for temperature extraction from thermal images at par with human raters with a tenfold reduction in time requirement. The automated process may replace the manual human extraction, leading to a faster process, making it feasible to carry out large-scale studies and to increase the regions of interest with minimal cost. The code for the developed algorithms, to extract the 44 ROIs from thermal images of hands, feet, and shins, has been made available online in the form of MATLAB functions and can be accessed from http://www.um.edu.mt/cbc/tipmid.

## 1. Introduction

Body temperature has long been used as a natural indicator of diseases [1]. Normally, the body maintains a constant core temperature of 37°C. The body naturally responds to changes in the peripheral regions, by sweating and other physiological processes, to control the core temperature. Diseases, such as peripheral arterial disease (PAD) and local infection, may affect this thermoregulatory process resulting in cooler extremities or increased peripheral temperature [2].

Peripheral arterial disease is a condition in which plaque builds up in the arteries carrying blood from the heart to the peripheries. Over time, the extent of plaque increases leading to narrowing of the lumen of the arteries, restricting blood flow to the peripheries and thus reducing the ability of the body to regulate the peripheral temperature [3]. Diabetic patients are more likely to suffer from PAD and its complications, with the lower limbs being the most affected area in what is commonly known as the diabetic foot [4]. Assessing the temperature in the lower limbs in patients suffering from PAD is critical to detect any arising complications, such as local infection, which may eventually lead to amputation of part of or the whole limb. Presently, the clinical practice of temperature assessment involves manual palpation of the lower limbs by the clinician. However, this method is unable to detect small temperature changes in the foot and may be affected by various exterior factors [2].

Thermal imaging detects the long-wave infrared radiation emitted from a surface of interest. The amount of radiation can be accurately related to the temperature of the same surface [5]. Human skin, with an emissivity value of 0.98, is a particularly suitable material for temperature measurement using thermal imaging [6]. This is reflected in the increase in the study and use of thermal imaging in medical and clinical applications [1, 7, 8]. One of the potential medical application areas is the monitoring of peripheral arterial disease, and its complications, in the diabetic foot [1, 9]. Compared to the present method of detecting differences in temperature in the diabetic foot using palpations of the plantar aspect of the foot, thermal imaging can detect very small changes in the surface temperature. Moreover, it can easily detect these changes in multiple places as opposed to the manual method which can monitor one region at a time. Furthermore, thermal imaging gives an objective value for the temperature in contrast with the subjective temperature estimate obtained from manual palpation.

Areas of increased temperature in the foot are predictive of the development of ulcerations. A difference in temperature in contralateral regions of the plantar aspect greater than 22°C is generally considered to be abnormal [10]. Benbow et al. have concluded that thermography is a simple, inexpensive, and noninvasive method for the identification of the neuropathic foot and the increased risk of ulceration [11].

Oe et al. found that when examining the thermal images in a case study of a subject with diabetes and a foot ulcer, a high-temperature area could be identified extending from the fourth toe (where the ulcer was present) to the ankle [12]. The patient was later diagnosed with osteomyelitis in the ulcer on the left foot. Based on these observations, thermography could prove useful for screening of foot ulcers for further complications. Tamaki et al. confirmed the predictive power of thermal imaging in detecting osteomyelitis in diabetic feet by studying 18 subjects with osteomyelitis and obtaining a 100% positive predictive value [13].

Nagase et al. [14] have monitored thermographic patterns in the plantar surface of the foot and compared the patterns between nondiabetic subjects and diabetic subjects. They have determined 20 different possible patterns which the plantar thermographic pattern can follow using the four different angiosomes in the plantar region of the foot. Both healthy control subjects and diabetic subjects were screened, and each of these was categorised to one of the 20 patterns. While the control group subjects were generally categorised in two of the patterns, the diabetic group had a larger distribution across the 20 patterns. This suggests that, by monitoring the thermal patterns in the plantar aspect of the foot, it is possible to identify any abnormalities and possibly detect complications in the diabetic foot.

In our previous work, Gatt et al. [2], we have determined the normative heat pattern distribution in hands, feet, and shins in a population of healthy adults. We identified 44 regions of interest (ROIs) across the three body regions from which the thermal patterns were established. Our aim was to repeat this study on a population of subjects which suffer from diabetes and PAD to identify any difference in the thermal patterns between the two populations. Such a study would help us to better understand the relationship between PAD and how it affects the peripheral temperature. However, such an analysis would require a large number of subjects. In the study of Gatt et al. [2], the temperature values were extracted manually by trained clinicians using an area demarcation software tool, namely, FLIR Altair software. This process was time-consuming, especially considering the large number of ROIs that had to be extracted. Additionally, regions extracted had a degree of subjectivity and nonrepeatability owing to the human element in the extraction process. An automated approach that allows for the rapid and consistent segmentation of regions of interest from thermal images is desirable as a first step in the monitoring of PAD using thermography. In this work, we will focus on the technical aspect of this segmentation process. Specifically, a method for automatic extraction of temperature data from ROIs on thermal images of human hands, feet, and shins is presented.

## 2. Related Work

The literature in the field of thermal imaging for the monitoring of diabetic feet, and medical thermography in general, focuses on the detection of abnormal regions of temperatures which may be indicative of emerging ulcerations or other pathologies. This is often done through asymmetry analysis in which regions from one foot are compared to the corresponding regions on the contralateral foot, with a deviation of more than 22°C being considered substantial to warrant further investigation [15–17]. In such studies, the body regions are segmented from the background such that corresponding contralateral regions can be compared. This step is generally carried out using either thresholding or clustering method [17, 18]. Once the body region is segmented from the background, the two contralateral regions are transformed such that corresponding pixels are aligned. This step is usually carried out using either rigid or nonrigid transformation based on automatically detected corresponding points of the body.

A further step beyond segmenting the body regions from the background is to subsegment the identified body regions. Yoon et al. [19] analysed thermal images of human arms, and automatically extracted the forearm region from the rest of the arm. Their method was shown to correctly extract the forearm regions; however, no further analysis was carried out on the segmented areas.

Herry [20] also segmented regions from thermal images of hands, with the fingers and the palm being identified. The subsegmented regions were visually inspected to provide qualitative results, but no quantitative performance measures were provided.

Blank and Kargel [21] proposed a method to identify finger and palm regions from thermal images of human hands using morphological opening with varying radii circular structuring elements. In an iterative process, the radius of the structuring element was increased and the area and circumference of the structure that remained after morphological opening were recorded. By detecting the two largest peaks in the first derivative of the change in area, *dA*/*dr*, the finger and palm regions could be identified. However, the derivative of the signal is susceptible to minor local variations and may result in false detections. Blank and Kargel tested their method on thermal images from seven subjects. When we tested this method on a larger test database, this method failed to reliably extract the correct points. In the work of Blank and Kargel, the average finger and palm temperatures were next computed from all pixels in these regions. The method is pose independent but critically depends on good background-foreground segmentation, as any areas which are segmented incorrectly will change the value and position of the peaks. Since in our work the hand thermal images have a good foreground to background contrast, this method was adopted with some variations to segment the fingers from the rest of the hand for subsequent ROI extraction.

The same method was also used by Gauci et al. [22] to subsegment the fingers and the palm from the rest of the hand in thermal images.

Gauci et al. [22] automatically extracted temperature values from anatomical regions of interest from thermal images of human hands, feet, and shins. The methods disclosed in this paper are an improvement of the algorithms proposed in this work.

Most of the reviewed studies stop at the background-foreground segmentation or at the subsegmentation stage, from which the mean temperature of the whole body region is computed [21]. However, Nagase et al. have shown that certain pathologies are characterised by localized variations in temperature [14]. A localized temperature variation might not substantially affect the average temperature of the whole body region, and thus the detectability of this local temperature variation is diminished if only the mean temperature is considered. Furthermore, the mean temperature of the whole body region does not provide any spatial information on the local temperature variations. Therefore in order to detect the pathology, temperature measurements need to be extracted from localized regions of interest rather than from the whole body region.

Scientific studies on the thermal patterns associated with normal and pathological subjects would benefit from automated image analysis techniques in order to allow for the processing of large volumes of data. Similarly, deployment of such tools in a clinical setting dealing with sizeable number of patients would benefit from automated techniques that would provide the physician with readily available results. This motivates the efforts towards automated techniques for the extraction of reliable temperature data from regions of interest. In this work, we extract consistent local regions of interest from the subsegmented body regions in order to measure specific local temperatures.

## 3. Methods

### 3.1. Data Acquisition

**Figure 1:**
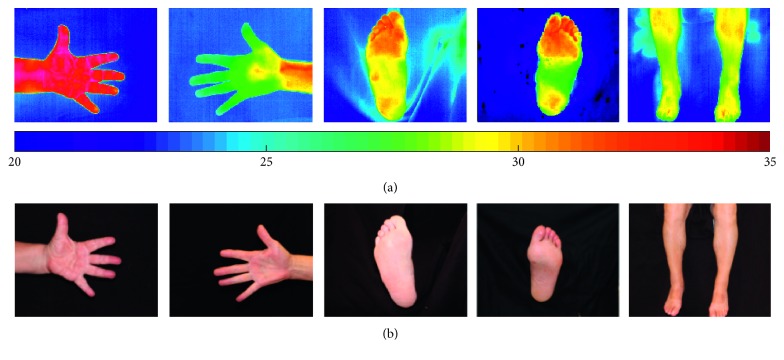
Sample thermal (a) and visual (b) images acquired in this study.

**Figure 2:**
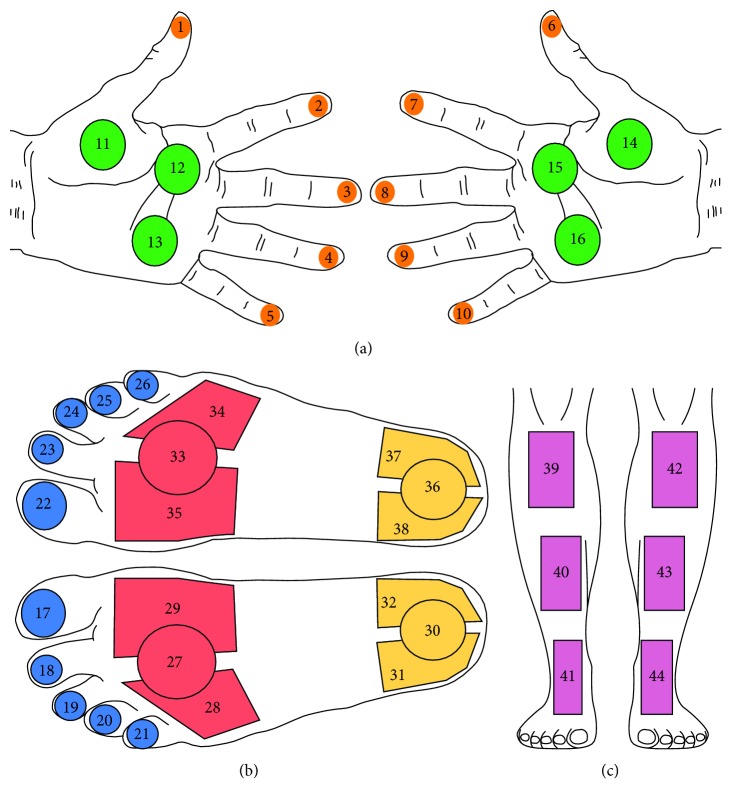
44 anatomical regions of interest identified on the hands (a), feet (b), and shins (c).

For the purpose of this work, thermal images of the volar aspect of hands, the anterior aspect of shins, and the dorsal aspect of the feet were considered. Thermal and visual images as shown in Figure 1 were acquired from each participant. A clutter-free background is assumed for the acquired images. In total, 44 anatomical regions of interest (ROIs) were considered as shown in Figure 2. These ROIs are linked to regions of the foot which are at higher risk of developing ulcerations, while the regions on the hands and the shins serve as baseline data as these regions are relatively unaffected by PAD. Since in our work, we are considering only healthy limbs without any amputated peripherals, we have a set of 44 ROIs for each subject that need to be found in the images and labelled uniquely with 44 distinct ROI labels. 395 subjects gave their informed consent to participate in this study which was approved by the University Research Ethics Committee at the University of Malta.

A FLIR SC7200M infrared thermal camera, which has a spatial resolution of 320 × 256 pixels and a thermal sensitivity of 20 mK with an accuracy of ±1°C, was used to acquire thermal images. Standard medical thermal image acquisition protocol was followed to acquire the thermal images [23, 24]. The camera was kept perpendicular to the surface being imaged and at a distance between 1.5 m and 2 m from the surface of interest. A corresponding visual image was acquired for every thermal image using a Canon EOS 1100D digital visual camera with a resolution of 12 MP. It is worth mentioning that the same anatomical portion of the corresponding body region was captured in all images. Specifically, in the hands, it is important to capture all the fingers and the palm and that these are centred in the image such that some of the forearm is also included. In the case of the shin images, the section of the shins from just below the knee till the toes has to be in full view and, in the case of the feet, the whole plantar aspect of the feet, including the toes, must be acquired. This is the only operator-dependent procedure in this algorithm.

Since the thermal and visual images were captured using different acquisition devices, images of the same region are not aligned. When capturing the images, the subject was asked to remain stationary during the acquisition of the thermal and visual images. Both images were captured from approximately similar poses and distances and perpendicularly to the surface of interest; thus, the two images are sufficiently close to orthographic and with little misalignment between the two images.

The segmentation methods developed herein exploit the shape and geometry of the body regions of interest. Since the body regions considered in this work vary significantly in shape, three different shape-based methods were developed, the hand images, the feet images, and the shin images, respectively.

### 3.2. Hands Segmentation and Extraction Algorithm

**Figure 3:**
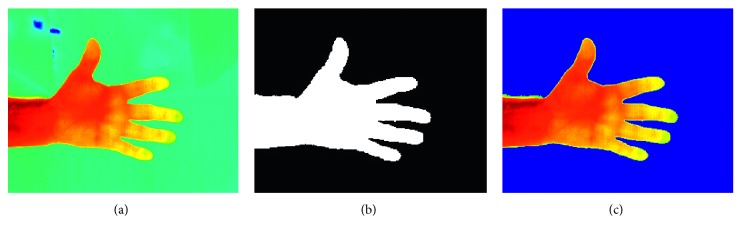
Segmentation of a sample hand thermal image using local adaptive thresholding. (a) Original thermal image. (b) Binary mask. (c) Segmented image.

The first step for every image, whether hand, foot, or shin, is that of identifying the body regions from the background in the thermal image. The hands usually have a significantly higher temperature than the background and consequently a higher intensity in the thermal image. However, regions at the edges of the hand, such as the finger tips, might have a significantly lower temperature than central regions such as the palm of the hand. For this reason, local adaptive thresholding for image binarisation was used so that the threshold is adapted locally for each pixel according to the intensity of the pixel neighbourhood. For the thresholding process, a Gaussian weighted mean statistic in a neighbourhood which is 1/16th the size of the image was used.  Figure 3 shows the segmentation of a sample hand thermal image.

**Figure 4:**
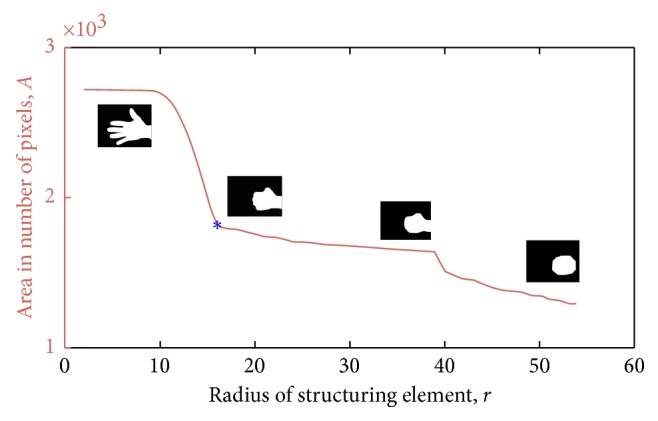
The number of remaining foreground pixels *A*, at each iteration with radius *r*, together with the resulting image, and the detected radius at which the finger removal process ends (blue).

**Figure 5:**
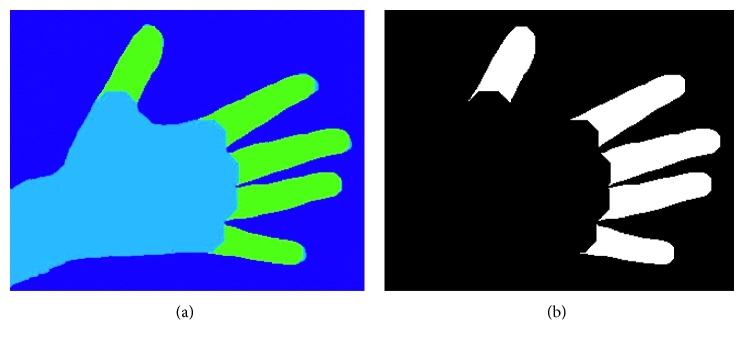
Identification of the finger pixels. (a) Detected finger pixels (green). (b) Fingers binary image.

Besides segmenting the hand from the background, it is necessary to identify the fingers. This was done using an adaptation of the iterative morphological opening approach proposed by Blank and Kargel [21]. Specifically a morphological opening operation using a disc-shaped structuring element (SE) was used to identify the finger pixels from the rest of the hand. The morphological opening operator was applied in an iterative manner, increasing the radius *r* of the disc-shaped SE at each iteration. For every iteration, the area *A*(*r*) of remaining foreground pixels was calculated.  Figure 4 shows the plot of *A*(*r*) versus the increasing radii of the SE, *r*, together with the actual remaining pixels on the image. Three salient features can be identified in the plot, namely, the sudden drop in area when the SE radius is large enough to remove the fingers from the image; the long plateau segment following this drop, where there is only minor smoothing of the palm region; and another sharp, but shallower, drop when the SE radius is large enough to remove the forearm from the image. The radius of the SE at the end of the first drop in area is used to identify the fingers from the rest of the hand. To avoid false detections arising due to the susceptibility of the first derivative, *dA*/*dr*, to local variations, the required radius was determined based on the variance of *A*(*r*) across the increasing *r* since this variance is less susceptible to local variations due to the averaging effect. Specifically, a variance measure *V*(*r*) at every point along *A*(*r*) was calculated. *V*(*r*) is defined as

(1) $$\begin{array}{c} V\left(r\right)=\frac{1}{N-1}\overset{r+N/2}{\underset{r^{\prime }=r-N/2}{\sum }}{\left(A\left(r^{\prime }\right)-\mu \left(A\left(r^{\prime }\right)\right)\right)}^{2}, \end{array}$$

where *N* is the size of a moving window around *r*. The midpoint of the signal *A*(*r*) was observed to generally lie on the second salient feature of the signal (i.e., the long plateau segment following the first drop; refer to Figure 4). Thus the midpoint of *A*(*r*) was used as a simple heuristic to provide a starting point for the process of identifying the required radius. Starting from this midpoint and moving towards *r*=0, the average variance of the first three neighbourhoods was used to establish a reference value. The first neighbourhood whose variance was greater than the reference by a predefined factor was taken to be the radius of SE required to identify the fingers. Using this radius, a binary image which contains only the fingers in the image can be obtained, as shown in Figure 5.

From the finger binary image, shown in Figure 5(b), each finger was identified by analysing the angle between the lines from the centroid of each finger blob to the centroid of the palm. The largest angle corresponds to the angle between the thumb and the little finger since these two fingers are the most further apart in the hand. Since each hand image is labelled as a left hand or a right hand, the direction of the angle between the two lines, clockwise or anticlockwise, can be used to distinguish the thumb from the little finger. The smallest angle from the identified thumb then corresponds to the angle between the thumb and the index finger. Using similar computations, all five fingers can be identified in a manner which is rotation independent.

**Figure 6:**
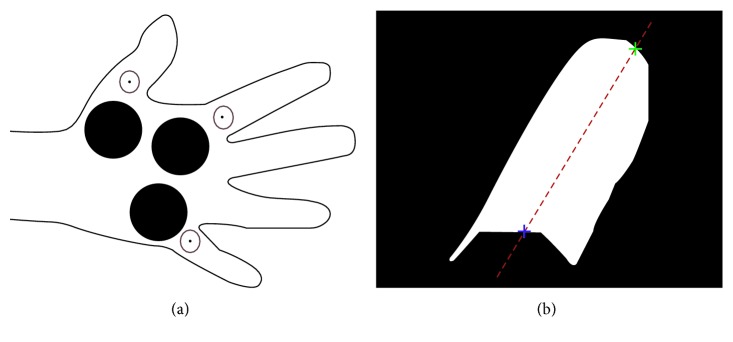
Palm ROIs extraction process. (a) Template used to extract the palm ROIs with three anchor points circled in red. (b) Detection of point corresponding to the anchor point on the thumb image. The blue intersection point is selected.

A template which contains the three ROIs on the palm of the hand was defined, as shown in Figure 6(a). The template has three anchor points, circled in red in Figure 6(a). To determine the 3 ROIs on the palm, the 3 corresponding points on the thermal image are determined by detecting the intersection points between the major axis of the thumb, index, and little finger blobs and the edges of the respective blobs, selecting the intersection point closest to the palm centroid. This process is shown in Figure 6(b), with the selected intersection point shown in blue. An affine transformation, mapping these three points to the corresponding anchor points on the template is determined to fit the template on the palm of the image being processed and determine the three ROIs on the palm.

**Figure 7:**
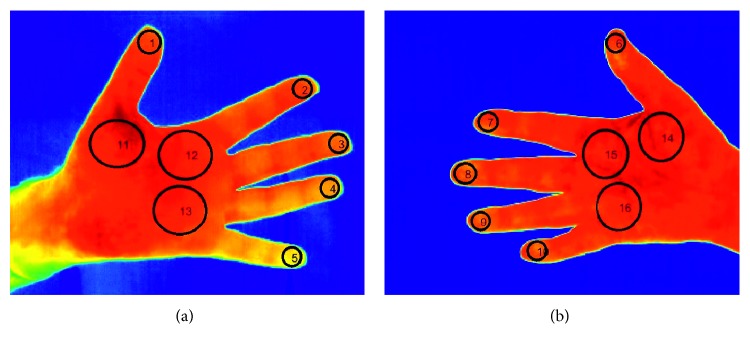
Identification of the ROIs on the hand.

To extract the five ROIs on the finger tips, the Hough transform was applied to the edges of each individual finger. If more than one circle is returned, the circle whose centre is closest to the fingertip of the finger, shown in green in Figure 6(b), is used and all other circles are discarded.  Figure 7 shows the hand ROIs extraction process.

### 3.3. Shins Segmentation and Extraction Algorithm

**Figure 8:**
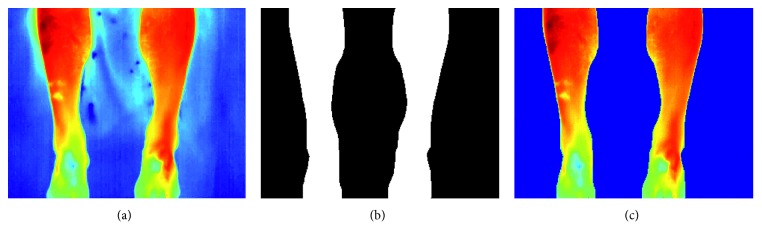
Segmentation of a shin thermal image using local adaptive thresholding. (a) Original thermal image. (b) Binarised thermal image. (c) Segmented thermal image.

Similar to the hands, the shins in the thermal images have a higher intensity than the background, and thus thresholding can be used to identify the shins from background. Local adaptive thresholding was once again used.  Figure 8 shows this procedure being applied on a sample shins thermal image.

**Figure 9:**
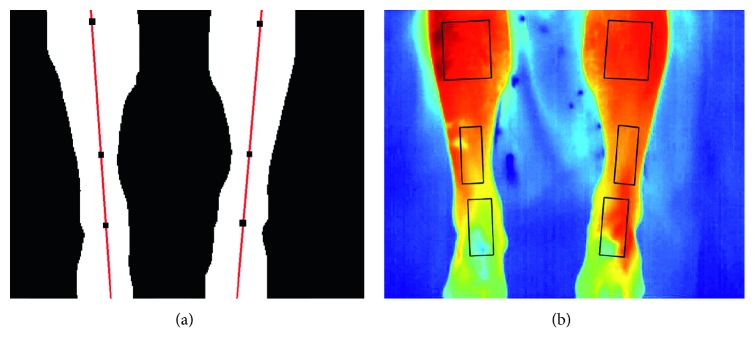
Shin ROIs extraction. (a) Extracted medial line (red) and identified centre points. (b) Identified and rotated rectangular ROIs.

Following binarisation, the medial line of each shin is found and the angle which this line makes with the horizontal axis of the image is computed. This angle is used as the orientation of the limb being processed. Three points are identified on the medial line which will serve as reference points for the placement of the ROIs on the shins. These points lie at the top of the shin, at the centre of the shin, and at 3/4 of the length of the shin according to the predefined position of the shin ROIs as shown in Figure 2. Each point is then used to place a rectangular ROI whose length and height are automatically scaled according to the local limb dimensions, which is rotated to follow the orientation of the limb being processed.  Figure 9 illustrates the process of extracting six rectangular ROIs on a sample thermal image.

### 3.4. Feet Segmentation and Extraction Algorithm

The temperatures in the feet are generally lower than the temperatures in the hands and the shins, and are usually close to that of the background temperature. Due to this lower temperature in the feet, there is a similarity in intensity between foreground and background regions in the thermal image making the background-foreground segmentation very challenging. On the contrary, the corresponding visual images offer a significant and consistent difference between the feet and the background and can be accurately segmented using Otsu's thresholding method. Therefore, the binarised visual image was used to segment the thermal image.

**Figure 10:**
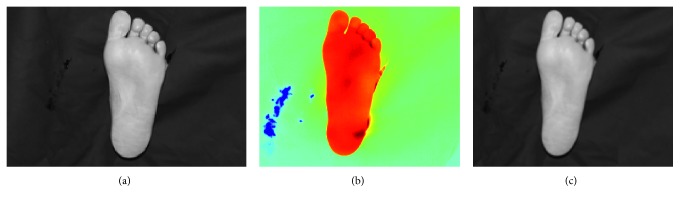
Alignment of the visual image to the thermal image. (a) Original visual image. (b) Original thermal image. (c) Aligned visual image.

**Figure 11:**
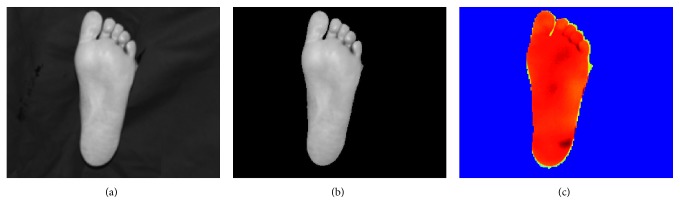
Segmentation of a thermal image of the foot. (a) Aligned visual image. (b) Segmented visual image. (c) Segmented thermal image.

The two images first had to be aligned. Since the visual and thermal images were captured almost simultaneously and close to perpendicular to the surface of interest, the difference between the two images mainly consists of rotation and scaling, with possibly some skewness. Thus, an affine transform is sufficient to align the two images. The visual image was aligned to the thermal image using an affine transformation based on, at least, three corresponding points on the visual and thermal images which were manually selected by the user.  Figure 10 shows the process of aligning a visual image to the corresponding thermal image.  Figure 11 illustrates the segmentation of a sample foot thermal image.

**Figure 12:**
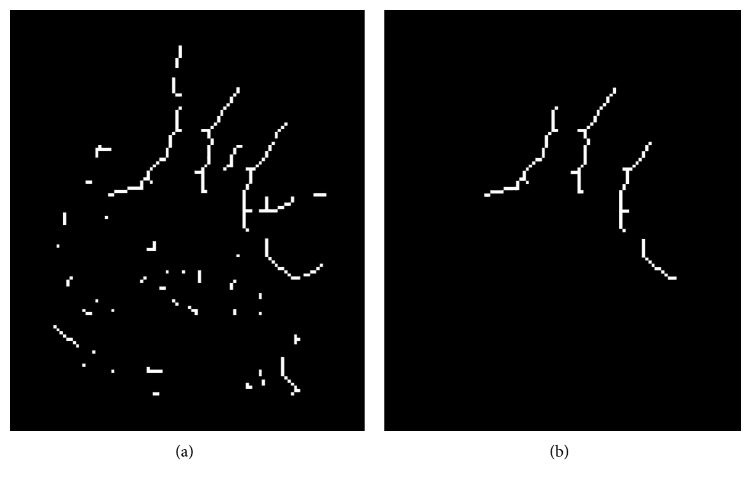
Extraction of edges close to the toes regions (a) and removal of unwanted edges (b). Remaining edges correspond to edge pixels in the regions between the toes.

The segmented foot thermal image was rotated such that the major axis of the foot, determined by eigenvalue decomposition, was vertically aligned. The aligned and segmented visual image was once again used in the extraction of the five ROIs on the toes. The edges around and between the toes were stronger and more consistent in the visual images than in the thermal images allowing for more reliable region extraction. A Laplacian of the Gaussian filter was used to extract the edges of the toes and nearby regions, in the upper half of the vertically aligned foot. The detected edges were binarised, skeletonised, and the outer contours of the foot were removed as shown in Figure 12(a). The resulting edge map contains both the edges around and between the toes, and spurious, unwanted edges arouse due to illumination changes and shadows on the foot in the visual image. In contrast to the edges around the toes, the unwanted edges are characterised by a lower intensity and a relatively smaller size. Consequently, these edges were removed by thresholding on the edge size and corresponding mean intensity, thereby retaining only the edges around and between the toes, as shown in Figure 12(b).

**Figure 13:**
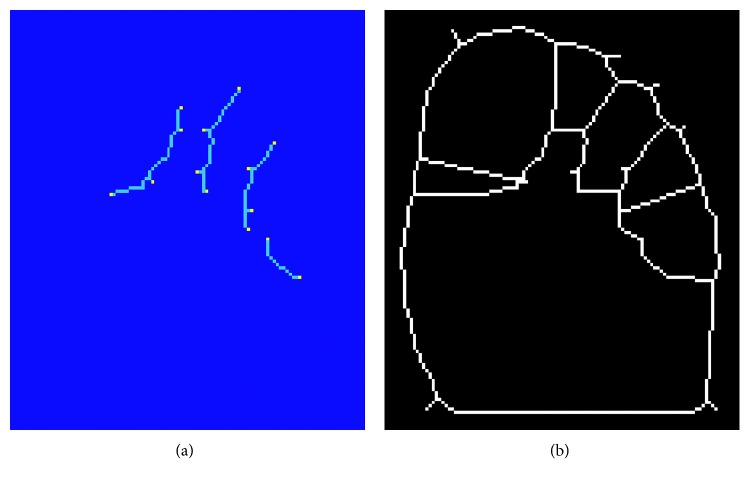
Detection of edge endpoint and connection of edges to form closed loops around the toes. (a) Detected edge endpoints. (b) Extended edges.

**Figure 14:**
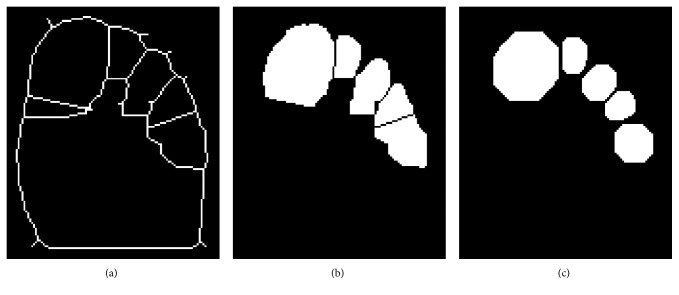
Regions completely contained within the extended edges are detected and shaped using morphological opening. (a) Extended edges. (b) Detected toes regions. (c) Disc-shaped regions.

The outer contours are next restored, and the resulting edge map is used to form closed contours around the toes. Edgels whose connectivity with their edge contours is through only one side are considered as endpoint edgels. These endpoint edgels were connected to the closest edgel in the edge map which is not on the same contour as the endpoint to form new contours as shown in Figure 13. The five largest closed regions detected in this manner are assumed to correspond to the toes, as seen in Figure 14(b). A morphological opening operator, using a disc-shaped structuring element, is applied to the resulting binary image to extract the remaining regions.  Figure 14 shows the results of this algorithm.

**Figure 15:**
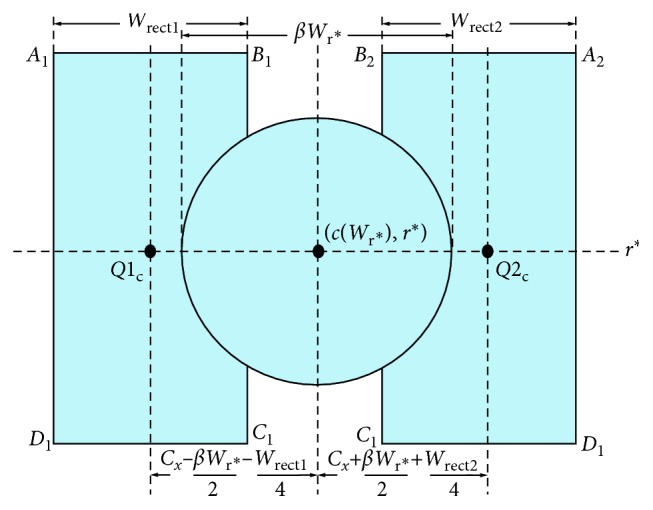
Positioning of the circular and quadrilateral ROIs on the ball of the foot and the heel.

To extract the circular regions on the ball of the foot (BOF), corresponding to regions 27 and 33 in Figure 2, an iterative process is applied which starts at the bottom row of the vertically aligned foot and moves up, towards the toes, one row at a time. In each iteration, a circle, *C*, is defined as

(2) $$\begin{array}{c} C=\left(c\left(W_{\text{r}}\right),r,\beta W_{\text{r}}\right), \end{array}$$

where *r* is the *y* coordinate of the circle centre, set to the current iteration row; *c*(*W*_r_) is the *x* coordinate of the circle centre, set to the midpoint of the horizontal width of the foot at row *r*, *W*_r_; and *βW*_r_ is the circle diameter where *β* is defined according to the desired size of the circular ROI according to Figure 2. The iterative process terminates at row *r*^*∗*^, when the Euclidean distance *d*(·) between the centre of circle *C*, denoted by *C*_*xy*_, and the middle row of the toe ROIs, *R*, satisfies

(3) $$\begin{array}{c} d\left(C_{xy},R\right)=\tau , \end{array}$$

where *τ* is set according to the position of the desired ROIs according to Figure 2. Once the candidate row, *r*^*∗*^, is chosen, two quadrilateral regions, regions 28-29 and 34–35 in Figure 2, are defined. Specifically, their centre points are set to

(4) $$\begin{array}{c} Q1_{\text{c}}=\left(C_{x}-\frac{\beta W_{{\text{r}}^{*}}}{2}-\frac{W_{{\text{rect}}_{1}}}{4},r^{*}\right),\\ Q2_{\text{c}}=\left(C_{x}+\frac{\beta W_{{\text{r}}^{*}}}{2}+\frac{W_{{\text{rect}}_{2}}}{4},r^{*}\right), \end{array}$$

where *C*_*x*_=*c*(*W*_r^*∗*^_) is the *x* coordinate of the circle centre and *W*_rect_1__=*W*_rect_2__=*αW*_r^*∗*^_ is the width of the quadrilaterals. *α* is set according to the desired size of the ROIs, as shown in Figure 2.  Figure 15 summarizes the placement procedure of the quadrilateral ROIs on the ball of the foot.

If there is any overlap between the extracted ROIs and the toe ROIs, vertices A and B in Figure 15 are moved horizontally downwards. If there is an overlap between the extracted ROIs and the background, vertices A and D are moved diagonally towards the quadrilateral centre. Since these ROIs are on the ball of the foot which typically has a convex shape, a diagonal movement is bound to remove the overlap whilst retaining the quadrilateral shape.

**Figure 16:**
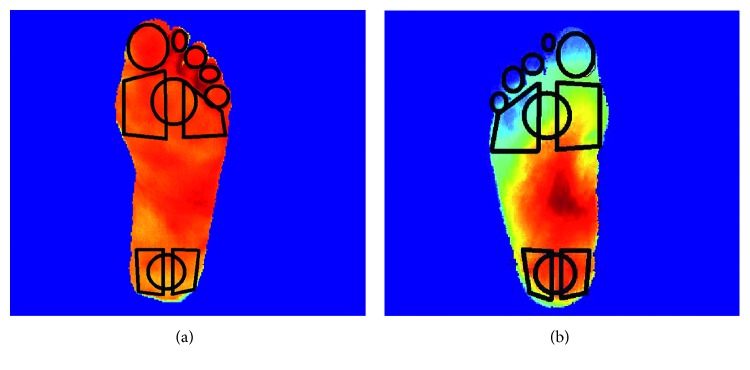
Identification of the ROIs on the feet.

To extract the three ROIs on the heel of the foot, corresponding to regions 30–32 and 36–38 in Figure 2, a similar procedure is carried out. The only difference to the extraction of the ROIs on the ball of the foot is the placement of the circle centre. To find the centre of the circle on the heel of the foot, the circle Hough transform is applied to the edges of the thermal image of the foot. Circles which have a diameter comparable to the width of the foot in the image are considered. The circle with the centre at the lowest position corresponds to the heel. This row position is also used to place the heel ROIs, and the same process used for the regions on the ball of the foot is repeated.  Figure 16 shows the results of extracting the 11 ROIs on feet thermal images.

The methods presented in this paper are publicly available, in the form of MATLAB functions, together with some sample thermal images and may be accessed from http://www.um.edu.mt/cbc/tipmid.

## 4. Results

### 4.1. Segmentation and ROIs Extraction

The algorithms were applied to the acquired thermal images, in a pipeline, one for each body region, and at every stage of the pipeline, a visual inspection was carried out to verify the integrity of the segmentation and the correct placement and sizing of the ROI labels. Based on this visual inspection, it was possible to take a decision to determine whether an ROI was extracted correctly or otherwise. The decision logic used for this purpose is as follows:

- A background-foreground segmentation was considered correct if it retained the integrity of the shape of the body region without any missing or additional features in the segmented image
- An extracted ROI was considered correct if it followed the correct placement and size with respect to the dimensions of the body region as defined in the template shown in Figure 2 and which does not overlap with the background regions or any other ROIs on the same image

One feature to be noted in the pipeline is that an error in one of the stages of the pipeline does not necessarily imply that the following algorithm will also fail.

The success rate was determined by obtaining the number of correct and incorrect ROI labels. In this study, a true positive (TP) would be a correctly localized ROI which is assigned its respective, distinct label; a false positive (FP) is an incorrectly localized ROI. Since, every image contains a specific number of ROIs and all ROI labels for an image are being assigned, negatives are undefined. Specifically, hand images contain eight ROIs, shin images contain six ROIs, and feet images have 11 ROIs, with each ROI having a specific ROI label.

As shown in Table 1, out of 155 hand images, 97.9% of the finger ROIs and 94% of the palm ROIs were correctly localized and labelled. The main source of failure was the background-foreground segmentation, which occurred in two hand images, and the misidentification of the fingers from the rest of the hand, which occurred in 11 other images.

As shown in Table 2, 99% of the shin ROIs were correctly localized and labelled. The main source of failure was the background-foreground segmentation which failed in three images due to a close similarity in temperatures between the foreground and background temperatures in the lower parts of the shins.

As shown in Table 3, 77.5% of the toe ROIs, 73.7% of the ball-of-the-foot ROIs, and 79.5% of the heel ROIs were correctly localized and labelled. The main sources of errors were the segmentation of the visual image, which failed in four images, and the misalignment of the thermal and visual images, which failed in eight images.

### 4.2. Method Validation via Interrater Reliability

To validate the segmentation results, an interrater reliability test was carried out. A set of 60 correctly segmented thermal images consisting of 20 hand images, 20 shin images, and 20 foot images were selected. Four human raters were asked to manually demarcate the ROIs using FLIR Altair area demarcation tool. The automated ROIs extraction method developed herein was used as the fifth rater in this study. The objective of this interrater analysis was to establish whether the results from the ROI extraction methods are comparable to the results obtained from human raters. The latter currently constitute the gold standard for region extraction. A spatial overlap coefficient, the Dice similarity coefficient (DSC) [25], was used to determine the similarity between the extracted regions. The DSC is defined as

(5) $$\begin{array}{c} \text{DSC}\left(A,B\right)=\frac{2\left|A\cap^{}B\right|}{\left|A\right|+\left|B\right|}, \end{array}$$

where *A* and *B* are the two target regions. The 44 ROIs were categorised into six groups as follows: fingers, palms, shins, toes, ball of the foot (BOF), and heel ROIs. The Dice coefficient was computed between corresponding regions between pairs of human raters and between the algorithm and the human raters. For each ROI group, there are six possible pairwise combinations between human raters, and four combinations between human raters and algorithm. The mean and standard deviation (SD) of the similarity coefficients for each group of ROIs was extracted for human rater pairs and for algorithm-human rater pairs as shown in Table 4.

Referring to Table 4, it is noted that, for all ROI groups, except for the toes, the mean algorithm-human rater coefficient is similar to the mean human rater coefficient. This implies that the algorithm may equivalently replace a human rater.

**Figure 17:**
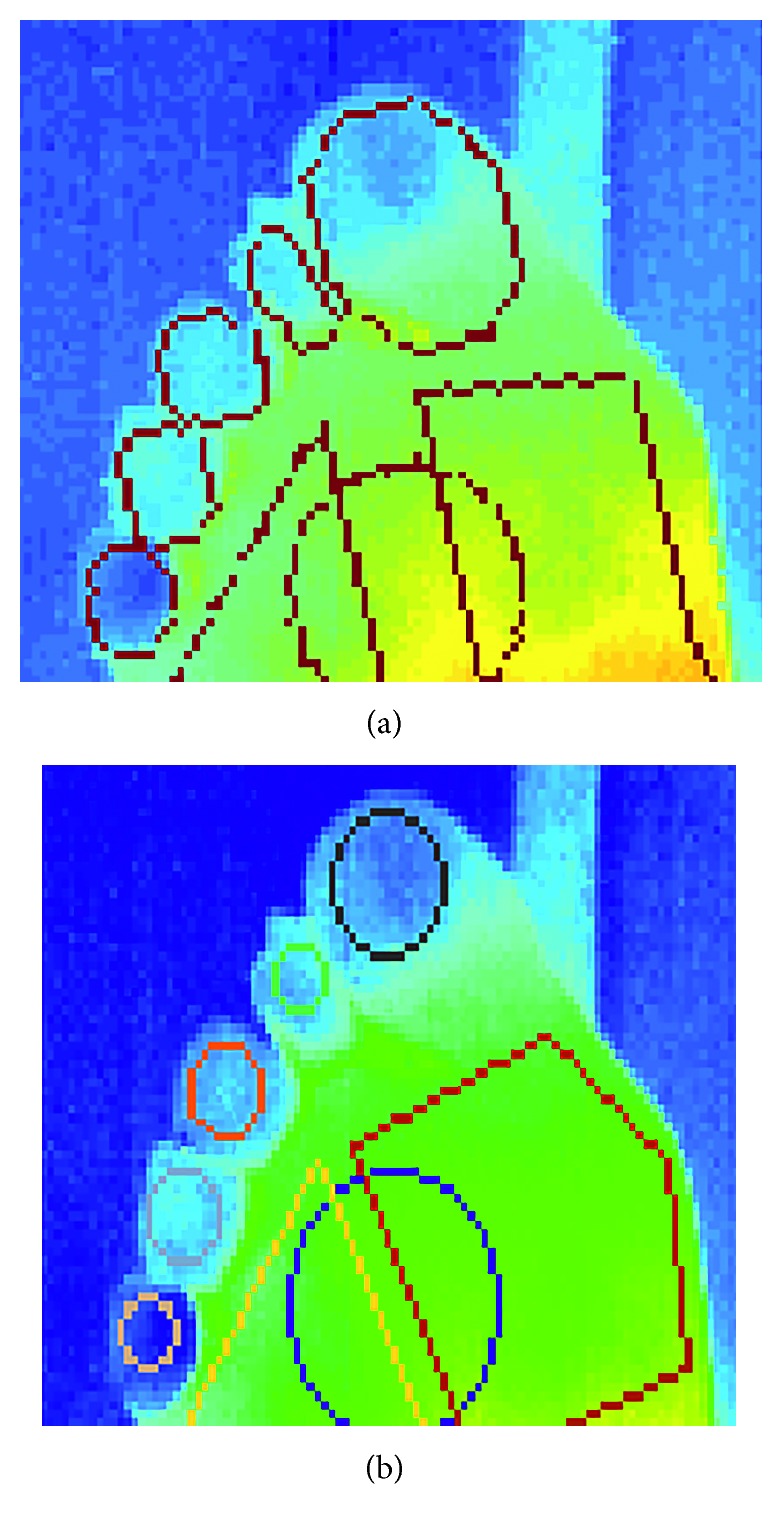
Comparison of automatic ROIs (a) and manual ROIs (b) extracted on the toes for the same image. Although the agreement between these regions is poor, the automatically extracted ROIs are more desirable.

For the toes group, a mean human rater coefficient of 0.621 (SD 0.182) and an algorithm-human rater coefficient of 0.427 (SD 0.170) were achieved, suggesting that the algorithm extracts ROIs that are substantially different than those extracted by humans. Through visual inspection of the ROIs was extracted by the algorithm, it was determined that the ROIs correctly cover the whole toe area, whereas the human rater ROIs are limited to circular ROIs, typically centred on the toes. Despite the discrepancy between the humans and the algorithm, it is noted that the algorithm ROIs are still valid and correct as shown in Figure 17.

Since the purpose of the ROI extraction is to measure the ROI temperature from the radiometric data, the mean temperature of the ROIs extracted by the algorithm was compared to that extracted by the human raters.  Table 5 shows the mean difference between the temperatures extracted by humans and by the algorithm. Since in the diabetic foot, abnormalities are associated with temperature differences of 2.2°C or greater, we considered a difference in temperature between the two sets of extracted ROIs of quarter of the 2.2°C reference as being negligible. A sign test confirmed that there was no statistically significant difference greater than 0.55°C at an alpha value of 0.05 between the mean temperature extracted by the algorithm and that obtained by the human raters, as shown in Table 5. This result demonstrates that the mean temperature extracted by the algorithm is, in substance, the same as that extracted by the human raters.

## 5. Discussion

The implemented algorithms were found to be effective for a large proportion of the thermal images on which they were tested. From Tables 1–3, it is noted that 484 ROIs required a manual intervention from a total of 3925 ROIs, which means an intervention in 12.3% of the ROIs. Therefore, 87.7% of the ROIs were extracted without any need for a manual intervention. The extraction of the foot ROIs was the most challenging out of the three algorithms due to the highly overlapping temperature ranges of the feet and the background in the captured thermal images.

Failed attempts at extracting the ROIs can be split in two categories: missed ROIs, in which the desired ROI has not been detected and there is no information about the temperature in the region, and inaccurate ROIs, in which the ROI is offset from the desired location.

When executed using MATLAB on a system with a 2.7GHz dual core processor and 8 GB of RAM, the algorithms required 1.4 seconds to process thermal images of the hands, less than 0.1 seconds for thermal images of the shins and 2.2 seconds for thermal images of the feet. Since for each subject, two hand thermal images, one thermal image of the shins, and two feet thermal images were acquired, and the algorithms required 7.3 seconds to process the five thermal images. The algorithm which processes thermal images of the feet also required a manual user intervention to register the thermal and visual images, which typically could be carried out in less than a minute. Therefore, the whole process of extracting the 44 desired ROIs from the five thermal images took around one minute to be completed. A corresponding process of extracting these regions manually, using an area demarcation tool, took an experienced user around 10 minutes. Therefore besides automating the area extraction process, significant reduction in the time required for the operation is achieved.

Interrater reliability tests showed that the ROIs extracted by the algorithm are within tolerance of the ROIs extracted by humans. This shows that the algorithms presented in this work can be used to extract temperature data with similar reliability and accuracy as humans.

## 6. Conclusion

Several medical applications of thermography require the extraction of temperature data from specific anatomical areas. Most literature in the field requires the clinicians to manually extract these data using area demarcation tools. This operation is subjective, may lead to nonrepeatable results, and is also very lengthy, especially for a large number of images and ROIs. Furthermore, the temperatures considered for such studies are averaged over large regions. This work proposes a set of algorithms to automate temperature extraction form local regions of interest. A success rate of around 90% was obtained for each algorithm, with minimal user intervention. In addition to the automation of the data extraction process, the algorithms also provide a significant reduction in the time required for the operation. The context for the algorithms is the detection of early signs of complications in the diabetic foot but can be extended to any application which requires temperature data from the same regions in the hands, shins, or feet. Future work should aim to enhance the algorithm to reduce the number of missed ROIs and to reduce the manual intervention required in the ROI extraction process.

## Figures and Tables

**Table 1:** Success rate of hand ROIs extraction.

Test database			
155 images	775 finger ROIs	465 palm ROIs	1240 total ROIs
Correct	759 (97.9%)	437 (94%)	1196 (96.5%)
Incorrect	16 (2.1%)	28 (6%)	44 (3.5%)

**Table 2:** Success rate of shin ROIs extraction.

Test database	
134 images	804 shin ROIs
Correct	796 (99%)
Incorrect	8 (1%)

**Table 3:** Success rate of foot ROIs extraction.

Test database				
171 images	855 toe ROIs	513 BOF ROIs	513 heel ROIs	1881 total ROIs
Correct	663 (77.5%)	378 (73.7%)	408 (79.5%)	1449 (77%)
Incorrect	192 (22.5%)	135 (26.3%)	105 (20.5%)	432 (23%)

**Table 4:** Dice similarity coefficients mean and standard deviation.

ROI group	Human rater pairs		Algorithm-human pairs	
	Size	Mean (SD)	Size	Mean (SD)
Fingers	600	0.756 (0.124)	400	0.688 (0.142)
Palms	360	0.698 (0.175)	240	0.691 (0.171)
Shins	720	0.552 (0.237)	480	0.512 (0.242)
Toes	600	0.621 (0.182)	400	0.427 (0.170)
BOFs	360	0.676 (0.192)	240	0.700 (0.148)
Heels	360	0.721 (0.134)	240	0.702 (0.128)

**Table 5:** Mean temperature differences and statistical test results.

ROI group	Algorithm-human differences	*ρ* value
	Mean (SD)	
Fingers	0.077 (0.079)	<0.02
Palms	0.159 (0.161)	0.023
Shins	0.364 (0.442)	0.08
Toes	0.145 (0.139)	0.09
BOF	0.132 (0.148)	0.007
Heels	0.117 (0.111)	0.007

## Data Availability

The methods presented in this paper have been made available online, in the form of MATLAB functions, together with some sample thermal images and may be accessed from http://www.um.edu.mt/cbc/tipmid.
